# Primary Hepatic Squamous Cell Carcinoma

**DOI:** 10.3390/diagnostics16010120

**Published:** 2026-01-01

**Authors:** Soo Ryang Kim, Soo Ki Kim, Hisato Kobayashi, Toyokazu Okuda, Yumi Fujii, Makiho Sakamoto, Yu-ichiro Koma, Osamu Nakashima, Motoko Sasaki, Akira Asai, Hiroki Nishikawa

**Affiliations:** 1 Department of Gastroenterology, Kobe Asahi Hospital, Kobe 653-0801, Japan; asahi-hp@arion.ocn.ne.jp (S.R.K.);; 2 Department of Radiology, Kobe Asahi Hospital, Kobe 653-0801, Japan; 3 Department of Surgery, Kobe Asahi Hospital, Kobe 653-0801, Japan; 4 Division of Pathology, Department of Pathology, Kobe University Graduate School of Medicine, Kobe 650-0017, Japan; 5 St. Mary’s Hospital, Laboratory Services Center, Kurume 830-8543, Japan; 6 Department of Human Pathology, Kanazawa University Graduate School of Medicine, Kanazawa 920-8640, Japan; 7 The Second Department of Internal Medicine, Osaka Medical and Pharmaceutical University, Takatsuki 569-8686, Japan

**Keywords:** imaging studies, de novo carcinogenesis, immunohistochemistry, primary squamous cell carcinoma of the liver, hepatic progenitor cell

## Abstract

**Background and Clinical Significance:** We present an 85-year-old male case of primary hepatic SCC manifesting as multiple liver nodules with atypical imaging findings. **Case Presentation:** The patient was negative for hepatitis B surface antigen and hepatitis C virus antibody. Serum tumor markers were all within normal limits. Contrast-enhanced ultrasonography with perflubutane demonstrated hypervascular nodules in the early vascular phase, early washout in the portal phase, and a defect in the postvascular phase (10 mm in S5 and 25 mm in S6). Histopathological examination revealed irregularly shaped tumor cells with large hyperchromatic nuclei and basophilic cytoplasm, surrounded by dense fibrous stroma forming cords, solid nests, and sheet-like structures. Immunohistochemical analysis showed positivity for AE1/AE3, p40, CK5/6, c-kit, and NCAM. **Conclusions:** The lesions were diagnosed as primary hepatic squamous cell carcinoma and suggested the possible involvement of hepatic progenitor cells, supporting the hypothesis of de novo carcinogenesis.

## 1. Introduction

Primary squamous cell carcinoma (SCC) of the liver is an exceptionally uncommon histopathological diagnosis. Only 32 cases have been reported in the English-language literature from the 1970s to date [1,2]. This low number of reported cases highlights the rarity of this malignancy and the consequently limited understanding of the disease [1].

SCC typically arises in tissues lined by squamous epithelium, including the skin, cervix, lungs, esophagus, and rectum. The liver parenchyma normally lacks squamous epithelial cells, making primary SCC in this organ exceptionally rare [1,3]. Primary hepatic SCC is often misdiagnosed [1,2,4]. Furthermore, its true histological origin remains a subject of controversy. Current reports suggest that this tumor may not arise directly from liver tissue; instead, it is thought to originate from the epithelial lining of developmental hepatic cysts, areas of chronic inflammation, or through the malignant transformation of the biliary epithelium [1,5]. Potential associations have been noted between hepatic SCC and conditions such as male gender, hepatic cysts, hepatolithiasis, solitary benign non-parasitic hepatic cysts (SBNHC), hepatic teratomas, and liver cirrhosis. However, cases have also been documented in patients without any preceding liver conditions or identified risk factors [5].

While a few cases of primary hepatic SCC have been treated successfully with initial surgery and chemotherapy, a standard therapeutic protocol for this disease has not been established [1]. This malignancy is considered aggressive, carrying an extremely poor prognosis; the median overall survival (OS) is reported to be less than one year [1,2].

Herein, we reported a case of primary SCC of the liver without chronic inflammation, preexisting hepatic cyst, hepatolithiasis, and hepatic teratoma.

And we also summarize and analyze the clinical features including imaging findings and histopathological findings, and discuss the mechanism of occurrence in primary SCC of the liver.

## 2. Case Report

### 2.1. Chief Complaints

An 85-year-old man was admitted to Kobe Asahi Hospital for further examination of multiple liver nodules. He had no fever, abdominal pain, or history of alcoholism.

### 2.2. History of Present and Past Illness

The patient presented with multiple liver nodules, but no other significant symptoms were noted. He had no history of chronic disease such as hepatitis or cirrhosis, and his past medical history was unremarkable.

### 2.3. Personal and Family History

The patient reported no significant family medical history. His personal history was negative for alcoholism or any known hereditary conditions.

### 2.4. Physical Examination

Physical examination upon admission revealed no significant findings. Jaundice, ascites and other clinical signs of liver disease were not found.

### 2.5. Laboratory Examinations

Laboratory tests upon admission revealed the following—total protein (TP) 6.4 g/dL (normal 6.5–8.3), albumin 2.9 g/dL (3.8–5.3), aspartate aminotransferase (AST) 40 IU/L (10–40), alanine aminotransferase (ALT) 26 IU/L (5–40), gamma-glutamyl transpeptidase (ɤ-GTP) 88 IU/L (<35), alkaline phosphatase (ALP) 70 IU/L (115–359), total bilirubin (BILI T) 0.4 mg/dL (0.2–1.2), NH3 15 μg/dL (<130), prothrombin time 85.6% (70–130), white blood cells 77 × 10^3^/μL (36–90), Hb 12.4 g/dL (11.5–15.0), platelets 19.3 × 10^4^/μL (13.4–34.9), hepatitis B surface antigen (-), hepatitis C virus antibody (-), antinuclear antibody (-), anti-mitochondrial antibody (-). Tumor markers: Alpha-fetoprotein (AFP) 6.1 ng/mL (<10.0), protein induced by vitamin K absence-II 36 mAU/mL (<40), carcinoembryonic antigen (CEA) 2.1 ng/mL (<5.0), carbohydrate antigen 19-9 (CA19-9) 12.7 U/mL (<37), prostate-specific antigen (PSA) 4.56 ng/mL (<4), SCC 1.0 ng/mL (<1.5), cytokeratin fragment (cyfra) 2.5 ng/mL (<3.5) (Table 1).

### 2.6. Imaging Examinations

**Figure 1:**
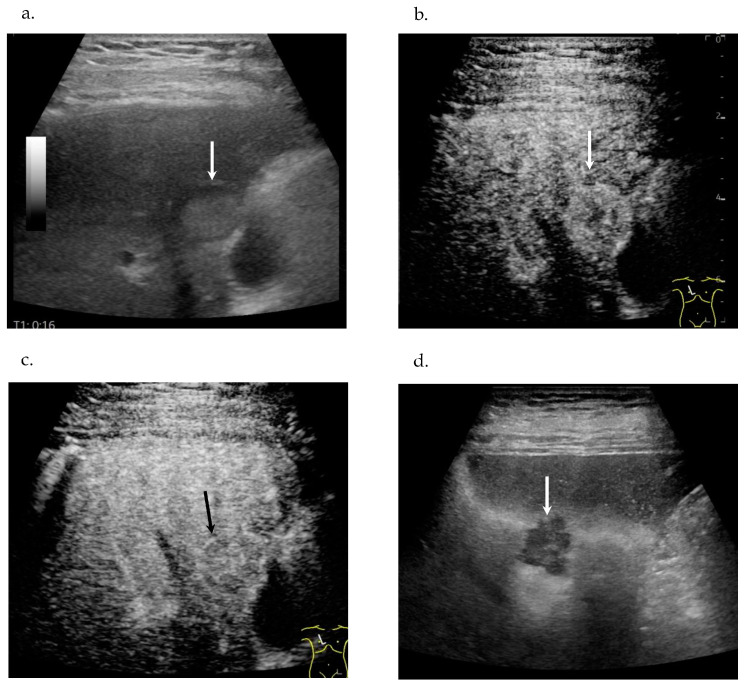

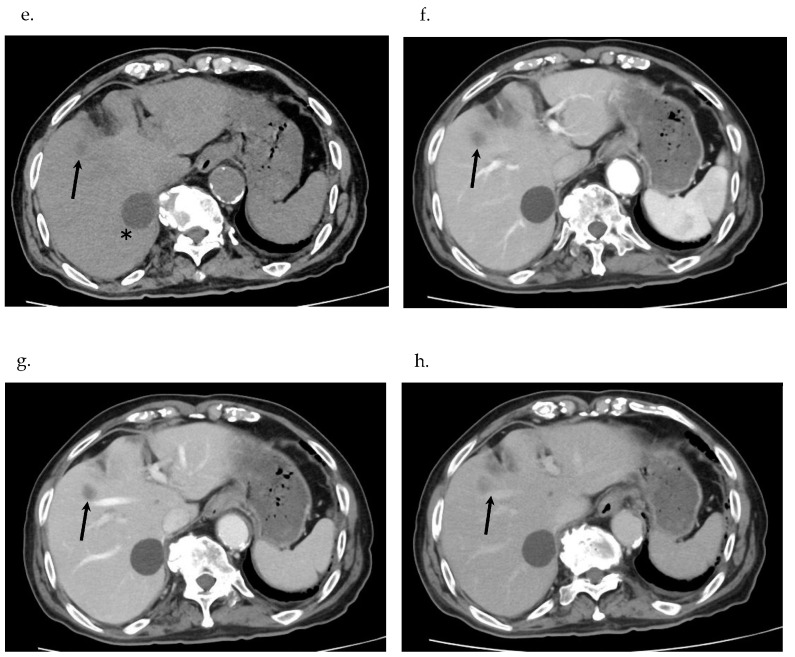
Ultrasonography (US). (**a**) B-mode Plain US, a 15 mm taro-shaped hyperechoic nodule with halo in S5 (arrow). (**b**) Contrast-enhanced US (CE-US), hypervascularity in the early vascular phase in S5 (arrow). (**c**) CE-US, early washout in the portal phase in S5 (arrow). (**d**) CE-US, defect in the postvascular phase in S5 (arrow). Computed Tomography (CT). (**e**) Plain CT, a 15 mm hypodense nodule in S5 (arrow) and a 45 mm hepatic cyst in S7 (asterisk). (**f**) Contrast-enhanced (CE-CT), hypovascularity in the early phase in S5 (arrow). (**g**) CE-CT, no enhancement in the portal phase in S5 (arrow). (**h**) CE-CT, the equilibrium phase in S5 (arrow).

Contrast-enhanced ultrasonography (CE-US), contrast-enhanced computed tomography (CE-CT), and magnetic resonance imaging (MRI) revealed the same nodules in S5, S6 and S8. US revealed a 10 mm hyperechoic nodule with a halo in segment 5 (S5) (Figure 1a) and a 25 mm taro-shaped hypoechoic nodule with a halo in S6.

**Figure 2:**
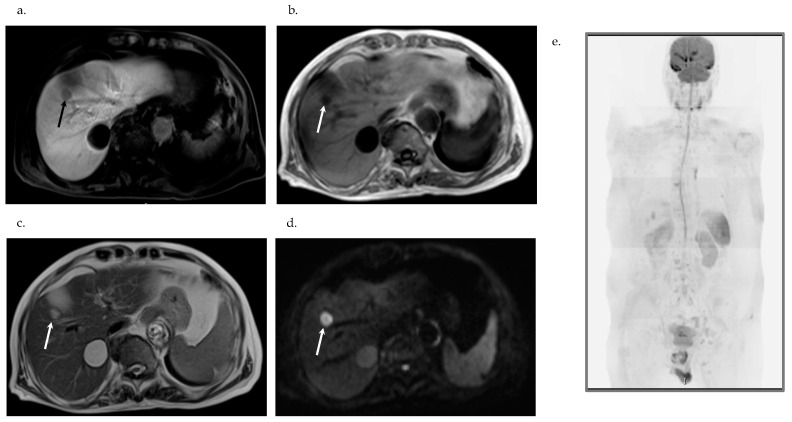
Gadolinium-ethoxybenzyl-diethylene-triaminepentaace-tic acid magnetic resonance imaging (Gd-EOB-DTPA MRI). (**a**) Defect in the hepatobiliary phase in S5 (arrow). (**b**) Hypointensity on T1W in S5 (arrow). (**c**) Hyperintensity on T2W in S5 (arrow). (**d**) Hyperintensity on DWI in S5 (arrow). Diffusion-weighted whole body Imaging with background body signal suppression (DWIBS). (**e**) Three hyperintense nodules only in the liver.

CE-US with the use of perflubutane revealed hypervascularity in the early vascular phase (Figure 1b), early washout in the portal phase (Figure 1c) and defect in the postvascular phase in S5 (Figure 1d) and S6. CT revealed a 10 mm hypodense nodule in S5 (Figure 1e), a 25 mm hypodense nodule in S6, a 10 mm hypodense nodule in S8, and a 45 mm hepatic cyst in S7 (Figure 1e). CE-CT revealed hypovascularity in the early phase (Figure 1f), no enhancement in either the portal phase (Figure 1g) or the equilibrium phase in S5 (Figure 1h), S6, and S8. Gadolinium-ethoxybenzyl-diethylene-triaminepentaace-tic acid MRI (Gd-EOB-DTPA MRI) revealed hypovascularity in the early phase and defect in the hepatobiliary phase (Figure 2a) in S5, S6, and S8, hypointensity on T1-weighted (T1W) (Figure 2b), hyperintensity on T2W (Figure 2c), and on diffusion-weighted imaging (DWI) (Figure 2d).

Plain CT revealed no tumor in the lung. Gastrointestinal fiberscopy (GIF) revealed no tumor in the esophagus or the stomach. Colonofiberscopy (CF) revealed a 21 mm polyp in the ascending colon. Complete endoscopic mucosal resection was carried out after histological analysis disclosed tubulovillous adenoma. Diffusion-weighted whole-body imaging with background body signal suppression (DWIBS) revealed three high intensity nodules only in the liver (Figure 2e).

US guided biopsy of the nodule in S5 disclosed irregularly shaped cells with large, hyperchromatic nuclei and basophilic cytoplasm at a high N/C ratio, dense fibrous stroma surrounding the tumor nest displaying cords, solid, and sheet-like structures.

### 2.7. Histopathological Examinations

**Figure 3:**
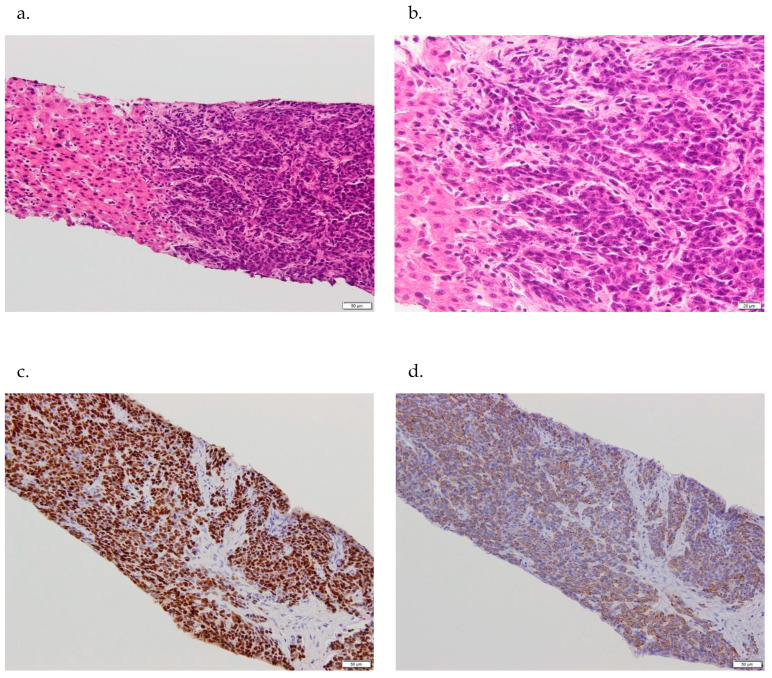

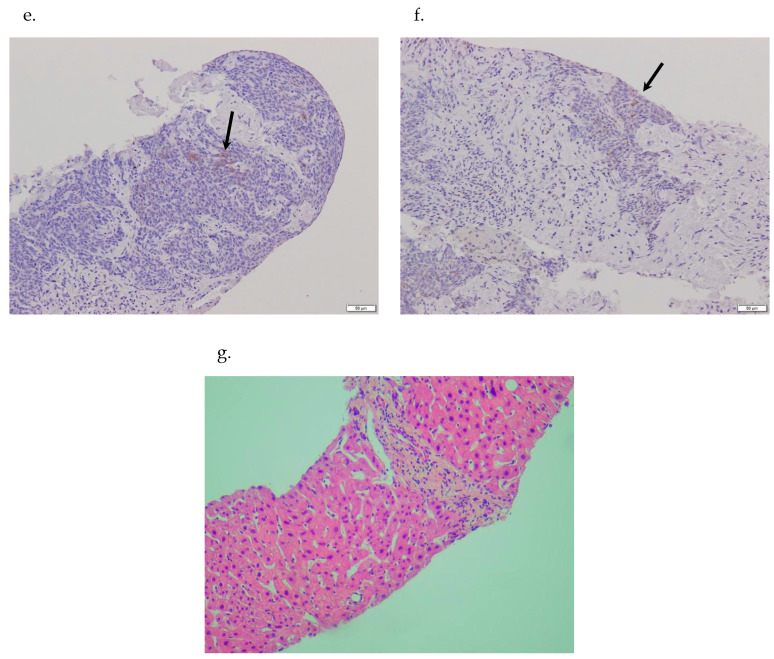
Pathological findings. (**a**,**b**) Irregularly shaped cells with large, hyperchromatic nuclei and basophilic cytoplasm with a high N/C ratio, dense fibrous stroma surrounding the tumor nest displaying cords, solid, and sheet-like structures. No distinct mucin-producing papillary or tubular structures are observed. Neither cancer pearl formation nor single-cell keratinization is in evidence. (**a**). (×200, HE staining), (**b**). (×400, HE staining). (**c**) Tumor cells positive for p40 (×200, immunohistochemical staining). (**d**) Tumor cells positive for CK5/6 (×200, immunohistochemical staining). (**e**) Tumor cells positive for c-kit (×200, immunohistochemical staining) (arrow). (**f**) Tumor cells positive for NCAM (×200, immunohistochemical staining) (arrow). (**g**) Non-tumor lesion. Very slight inflammation in the portal area but no fibrosis (×100, HE staining).

Inflammatory cell infiltration was observed around the tumor nest. No distinct mucin-producing papillary or tubular structures were found. Neither cancer pearl formation nor single-cell keratinization was observed. Poorly differentiated or undifferentiated carcinoma was suspected (Figure 3a,b). Immunohistochemical examination of tumor cells revealed positive reaction to AE1/AE3, CK20, protein (p)40 (Figure 3c), cytokeratin (CK)5/6 (Figure 3d), c-kit (Figure 3e), and neural cell adhesion molecule (NCAM)36 (Figure 3f) and negative reaction to GATA3, PSA, CK-7, CK-8, synaptophysin, and chromogranin A. A non-tumor lesion revealed very slight inflammation but no fibrosis in the portal area (Figure 3g).

## 3. Multidisciplinary Expert Consultation

A multidisciplinary team, comprising radiologists, pathologists, and hepatobiliary specialists, discussed the case. Based on the imaging findings and pathology, a diagnosis of primary SCC of the liver was considered, with differentiation from other liver tumors, such as hepatocellular carcinoma (HCC) and intrahepatic cholangiocarcinoma (ICC).

## 4. Final Diagnosis

The final diagnosis was primary SCC of the liver, based on pathological findings, immunohistochemistry and clinical correlation.

– Radiological findings ruled out HCC. Therefore, ICC, mixed HCC and ICC tumors, and metastatic tumors need to be differentiated.
– Based on pathological findings, the liver tumor was diagnosed as poorly differentiated SCC. Clinically, no primary focus suggesting a metastatic liver tumor in other organs was in evidence; based on GIF, CF, CT and DWIBS, the tumor was finally diagnosed as primary SCC of the liver. Also clinically, the nodule in S6 was a primary SCC lesion and multiple nodules located in S5 and S8 were interpreted as the result of intrahepatic spread from one of the primary SCCs of the liver.

## 5. Treatment

The patient declined chemotherapy and surgery.

## 6. Outcome and Follow-Up

The patient died 5 months after the diagnosis and without treatment.

## 7. Discussion

### 7.1. Epidemiology

Primary hepatic SCC is a rare malignancy, often posing a diagnostic challenge until histological confirmation is achieved. Data from a published case series indicate that patient ages span a broad spectrum, from 18 to 83 years, with a nearly equal male-to-female ratio of approximately 19:16 [1].

### 7.2. Symptoms and Examinations

Clinical presentation is highly variable, often including non-specific symptoms such as abdominal pain, jaundice, weight loss, reduced appetite, and difficulty swallowing [2].

Primary hepatic SCC patients typically exhibit abnormal liver function tests, marked by elevated levels of ALT, AST, total bilirubin (BILI T), and direct bilirubin (BILI D) [4]. This impaired function is understood to be linked to chronic inflammation within the bile ducts, the presence of hepatic cysts, or obstruction of the bile duct caused by tumor invasion or mass effect [2]. However, it is important to note that patients with primary hepatic SCC are not invariably found to have pre-existing liver damage or established risk factors [5].

Alpha-fetoprotein (AFP) levels have been observed to be within the normal range in all reported patients. Conversely, increased levels of CEA, CA125, and CA19-9 are frequently detected, though these markers are not specific for this diagnosis. Leukocytosis is present in about 30% of cases, often without associated fever, a finding that could potentially be explained by tumor-related leukocytosis (TRL) [6].

In our case, there were no symptoms such as abdominal pain, jaundice, dysphagia, weight-loss, decreased appetite, and fever. Laboratory data showed the normal range of ALT, ASL, and BILI T. ɤ-GTP showed high value, and TP and albumin showed low value. Tumor markers related to HCC such as AFP, PIVKA 2, related to CCC such as CEA, CA19-9, and related to SCC such as cyfla were within normal limits. Only PSA related to prostate cancer showed a higher value exceeding the normal range.

### 7.3. Imaging

Imaging studies, particularly enhanced CT and MRI, are essential for localizing the primary tumor. They provide valuable data on lesion count, dimensions, tumor extent, invasiveness, the presence of biliary dilation, and the potential for complete resection [1,3].

CT imaging typically shows heterogeneous, low-density masses. These lesions are sometimes found in conjunction with hepatic cysts or stones within the intrahepatic bile ducts. After contrast injection, the majority of cases demonstrate either rim or delayed enhancement, a pattern resembling that of intrahepatic cholangiocarcinoma (ICC). Nonetheless, the overall imaging characteristics of primary hepatic SCC are often atypical. Contrast-enhanced MRI (CEMRI) frequently reveals non-specific findings, such as peripheral and irregular enhancement in the arterial phase, with similar enhancement characteristics persisting into the portal and delayed phases [7,8,9,10,11,12]. Contrast-enhanced ultrasound (CEUS) typically shows heterogeneous enhancement during the arterial phase, followed by a washout effect in the late phase—a feature commonly associated with other malignancies like ICC [13].

Given the variability in enhancement patterns across CEUS, CECT, and CEMRI, primary hepatic SCC should be included in the differential diagnosis alongside ICC and vascular liver tumors [14].

The tumor mass usually contains a large, unenhanced central necrotic area with irregular inner margins and occasional nodular projections of varying sizes. Importantly, this tumor is typically not linked to underlying chronic liver diseases like cirrhosis [1,2]. At least for HCC, multilocular emergence or carcinogenesis is better than intrahepatic spreading. In case of another kind of liver tumor such as ICC, however, intrahepatic spreads rather than multilocular emergence or carcinogenesis are usually seen as metastatic mode. When it comes to the primary SCC of the liver in our case, intrahepatic spreads might have occurred like in ICC.

Differentiation from liver metastasis and liver abscess is necessary. In addition, SCC of unknown primary was not denied in our case. CT revealed no tumor in the liver. We performed DWIBS and confirmed three high intensity nodules only in the liver. No high intensity nodules were observed in the whole body. We also performed gastroendoscopy and colon fiberscopy and confirmed no SCC tumor in the esophagus, the stomach, and the colon. From the above findings, we diagnosed our case as primary SCC of the liver.

Imaging studies of our present case negated HCC, which needs to be differentiated from ICC, mixed HCC, and metastatic carcinoma. Primary hepatic SCC could not be diagnosed solely from the above imaging findings. Diagnosis needs to be confirmed through biopsy.

### 7.4. Histopathology

Poorly differentiated cholangiocarcinoma with squamous differentiation, adenosquamous carcinoma with sampling bias, or SCC from an occult primary site should be differentiated in our case.

Regarding histopathological findings including immunohistochemistry, Lee H.L. et al. reported that histological examination (H&E staining) of liver SCC biopsy specimens showed that the tumor cells were arranged in nests and displayed features of abnormal nuclear morphology and keratinization, including the formation of keratin pearls. Immunohistochemistry confirmed the tumor cells were positive for p63 and p40. Since CK7 is known as a marker for biliary epithelial cells, the co-positivity for CK7, p63, and p40 in their case study supports the histological diagnosis of SCC and implies a potential link to malignant transformation originating from the biliary tract epithelium [15].

Xiao J. et al. noted that the typical immunohistochemical profile of primary hepatic SCC includes positivity for p40, CK5/6, and p63. They also highlighted that CK19 and CK7, which mark glandular duct structures, are useful for distinguishing primary SCC from neoplastic cells derived from bile duct precursors. Hepatocellular carcinoma (HCC) is typically identified by markers such as arginase-1, GPC3, HepPar-1, and AFP, while cholangiocellular carcinoma (CCC) is positive for CK19 and CK8. In the cases they reported, all patients stained positive for p63 but were negative for CK7, HepPar-1, and CK19. The patient testing positive for CK5/6 suggested that the cancer cells might originate from the basal cells of the keratinized squamous epithelium. Furthermore, a primary tumor focus outside the liver (e.g., lungs, thyroid, gastrointestinal tract) was excluded using a combination of clinical, radiological, endoscopic, and endocrine evaluations [2].

Benhamdane A. et al. emphasized that a liver biopsy is necessary for definitive diagnosis. Immunohistochemically, the strong positive staining for CK14 and CK56 suggests that the cancer cells originated from basal cells of squamous keratinized epithelium. Additionally, the positive expression of CK19 confirms the biliary ontogeny of the neoplastic cells [12,16].

In our study, histopathological findings with only HE staining suggested poorly differentiated or undifferentiated carcinoma that needed to be differentiated from prostate carcinoma, urinary tract carcinoma, squamous cell tissue and neuroendocrine carcinoma. We think that keratin pearl formation or single-cell keratinization is related to the grade of cancer differentiation. Keratin pearl formation or single-cell keratinization is usually observed in well differentiated SCC. Poorly differentiated SCC in our case showed no findings of keratin pearl formation or single-cell keratinization in HE staining. Immunohistochemical analysis disclosed positive staining to AE1/AE3, CK20, CK5/6, P40, c-kit and NCAM, and negative staining to CK-7, CK-8, AFP, GATA3, PSA, synaptophysin, and chromogranin A. Thus, the tumor of epithelial cell origin was confirmed through AE1/AE3 positive staining. The negative staining of PSA ruled out prostate carcinoma, as well as urinary tract carcinoma by the negative staining of GATA3.

The negative staining of synaptophysin and chromogranin A ruled out neuroendocrine tumors. The significance of the positive staining for NCAM as one of the neuroendocrine tumor markers is discussed later in the paper together with the positive staining of c-kit. Positive staining of CK5/6 and P40 indicated the carcinogenesis of squamous cell carcinoma. Exclusion of the carcinogenesis of squamous cell carcinoma from other organs, such as the lungs, thyroid, and esophagus is a prerequisite to the diagnosis of primary SCC of the liver. Several primary hepatic SCC cases with multiple nodules were described in references No. 2 (Xiao J. et al.), No. 14 (Zhao L. et al.), and No. 15 (Lee H.L. et al.) [2,14,15]. The authors did not describe, however, the histopathology of multiple nodules respectively. They described the only representative histopathology of one nodule. In our case, squamous carcinoma from sites other than the liver was ruled out clinically, radiologically, and/or endoscopically; finally, the tumor was diagnosed as primary SCC of the liver.

### 7.5. Etiology and Pathogenesis

The etiology and pathogenesis of primary SCC remain largely unclear. Common risk factors for other primary liver tumors, such as Hepatitis B or C infection or cirrhosis, do not show a consistent association with the development of this disease [8,13,17]. Nevertheless, a possible link between the presence of hepatic cysts or hepatolithiasis and the incidence of primary hepatic SCC should not be dismissed [7,8]. The main proposed mechanism is chronic inflammation caused by the infection of hepatic cysts, or by irritation of the bile ducts due to stones, teratomas, or cirrhosis, which can induce squamous metaplasia and subsequently lead to SCC transformation in certain instances [7]. However, our current case lacked evidence of these pre-existing hepatic lesions or biliary abnormalities.

Hepatic cysts are lined by squamous epithelium, which is believed to undergo a progressive process of dysplasia, metaplasia, and eventual malignant transformation over many years [18]. Despite these proposed pathways, the exact molecular steps that culminate in carcinoma development remain undefined.

Although most cholangiocellular carcinomas are histologically adenocarcinomas, SCC of the biliary system is exceptionally rare, and its specific characteristics are not fully elucidated. The initial report detailing a case of primary hepatic SCC with a clear histological collision of adenocarcinoma and SCC has been published [19].

In our case, no histological collision with adenocarcinoma and SCC was observed.

Primary liver cancers (PLCs) exhibit a wide range of features, including hepatocytic, cholangiocytic, or mixed differentiation. This high degree of tumor heterogeneity is attributed to the liver’s four distinct epithelial cell types (hepatocytes, mucin-producing or non-mucin-producing cholangiocytes, and hepatic progenitor cells (HPCs)), which can give rise to PLCs in the context of chronic liver diseases with various etiologies [20,21,22].

The association between the cancer cell of origin (CCO) and the cells responsible for tumor propagation, referred to as hepatic cancer stem cells (HCSCs), has yet to be fully defined. Identifying a specific phenotypic marker for HCSCs has proven difficult, likely because hepatocellular carcinoma (HCC) contains diverse subpopulations of cancer stem cells (CSCs), each with unique functions. A dynamic interconversion occurs between various CSCs, as well as between CSCs and non-CSCs. Consequently, the CSC-state is now viewed not as a static tumor subpopulation but as a highly adaptable and intrinsic property of the tumor cells themselves. The acquisition of stemness can be triggered by various changes, including EMT-MET transition, epigenetic modifications, the tumor microenvironment, and selective pressures like chemotherapy. This inherent heterogeneity and dynamism of CSCs limit the effectiveness of therapeutic strategies that target a single population. Future research should focus on elucidating and targeting the underlying mechanisms that drive this interconversion of tumor cell populations [17,23,24,25,26].

Multipotent stem/progenitor cells are found in the peribiliary glands of extrahepatic bile ducts across all human ages, with the highest concentration in the hepato-pancreatic common duct, cystic duct, and hepatic hilum. These cells intranuclearly express endodermal transcription factors (e.g., Sox9, SOX17, FOXA2, PDX1, HES1, NGN3, PROX1) and various stem/progenitor surface markers (EpCAM, NCAM, CD133, CXCR4). They may also show weak expression of adult liver, bile duct, and pancreatic-associated genes (e.g., albumin, CFTR, insulin) [27].

Small hepatic parenchymal cells located outside the portal tract that are positive for biliary-type cytokeratins are believed to represent hepatic stem cells, the Canals of Hering (CoH), and/or remnants of the ductal plate. C-kit positivity was found in normal CoH [28].

Based on the above findings, two factors were attributed to the occurrence of SCC in our case.

(1) Transformational carcinogenesis; when the tumor transforms from adenocarcinoma of CCC, despite the absence of morbidity of the bile duct, inflammation and cysts. A 45 mm hepatic cyst was observed in S7, and a nearly 10 mm SCC tumor in S8; however, a hepatic cyst in S7 is considered unrelated to SCC tumors. Also, no adenocarcinoma component was observed in the biopsied specimen.
(2) De novo mode carcinogenesis; primary SCC of the liver can develop from hepatocytes or intrahepatic cholangiocytes de novo. The tumor might originate from hepatic progenitor cells or hepatic stem cells. Immunohistochemically, positive staining for NCAM and c-kit, characteristics of hepatic progenitor cells support the above hypothesis irrespective of the limitation on small biopsies in our case [27,28].

We could not perform, however, the immunohistochemistry of additional informative markers such as P63, ck14, SOX9, EpCAM due to scant biopsied specimen and molecular profiling. This weakens both diagnostic and pathogenetic conclusions.

Testing positive for c-kit and NCAM alone is insufficient to support de novo carcinogenesis, as these markers are not specific for hepatic progenitor cells and may be expressed in poorly differentiated carcinomas of various origins. Without molecular, spatial, or functional validation, de novo carcinogenesis should be presented more cautiously in our case.

### 7.6. Treatment

Given the extreme rarity of this entity, a standardized treatment protocol is lacking, and there is currently no universally accepted regimen for hepatic SCC. The available data is restricted to individual case reports, which describe patients who underwent aggressive interventions such as surgery or chemotherapy, often resulting in limited overall survival. Nevertheless, the only approach that appears to confer a survival advantage is radical surgery performed with curative intent, which involves achieving negative margins during liver resection, consistent with standard oncological surgery principles [7,8,13]. Less invasive treatments, such as cyst drainage or partial cystectomy for a complex cystic mass, must be approached with caution. A high index of suspicion is paramount and represents the initial step toward an appropriate diagnostic and therapeutic pathway. Evidence supporting the oncological efficacy of locoregional therapies, including thermal ablation, cryoablation, radiotherapy, and transarterial chemoembolization (TACE), remains limited for disease control [3,13,18,29].

Boscolo et al. documented a favorable response using neoadjuvant chemotherapy with cisplatin and 5-FU for tumors initially deemed unresectable [30].

Patients who received surgery or interventional treatments (like TACE and ablation) were categorized into the surgery or intervention groups, respectively. The non-operative group consisted of patients treated with radiotherapy, chemotherapy, immunotherapy, or a combination thereof. It is worth mentioning that treatment details were unavailable for some patients.

With respect to immunotherapy using PD-1 or PD-L1 inhibitors for primary hepatic SCC, Chan KK et al. reported a case where genetic testing revealed PD-L1 Tumor Proportion Score (TPS) of 1%, a Tumor Mutational Burden (TMB) of 24.93 mutations/Mb, a Genomic Profiling Score (GPS) of 1, and Microsatellite Instability-High (MSI-H). Given that pembrolizumab (a PD-1 inhibitor) is approved for various cancers with TMB ≥10 mutations/Mb or MSI-H, it was administered in this case. The TPS and GPS results provided additional rationale for this treatment choice [31]. Zhao L. et al. described a 60-year-old female patient whose lesion size dramatically decreased (from 11.9 × 9.4 cm to 4.9 × 3.9 cm) after one year of treatment with pembrolizumab (100 mg on day 1) and albumin-bound paclitaxel (400 mg on day 2) every 21 days (q21day), thus illustrating the therapy’s effectiveness [14]. The combined approach of surgery with PD-1 inhibitors has been associated with consistently better outcomes when feasible [11,14,31].

Zhang B. et al. noted that immunotherapy utilizing PD-1 or PD-L1 inhibitors has received approval for various advanced human cancers, such as SCC of the lung, esophagus, skin, and head and neck [32].

Conversely, Xiao J. et al. observed that two patients exhibited positive PD-L1 expression for tumor cells, and one patient tested positive for immune cells, suggesting potential activation of the PD-1/PD-L1 pathway. Despite the lack of established clinical protocols for primary hepatic SCC, PD-1 inhibitors that block the PD-1/PD-L1 pathway could emerge as promising agents by stimulating the host’s anti-cancer immune response [2].

Despite the scarcity of cases, further research is warranted to solidify the evidence base for immunotherapy’s efficacy in primary SCC.

### 7.7. Prognosis

The outlook for primary hepatic SCC (PSCC) remains exceedingly poor, as demonstrated by the fact that many individuals succumb to the disease shortly after diagnosis. The prognosis is unfavorable, and the patient’s mean survival duration is reported to be under 12 months [1,2]. A literature review spanning from 1997 to 2016 indicated that the median overall survival (OS) and disease-specific survival (DSS) for this patient group were 7.7 months and 2.0 months, respectively [2].

The factors that determine prognosis are still unclear. However, radical surgery remains the cornerstone of therapeutic recommendations. Other therapeutic options, including chemotherapy, arterial embolization, tyrosine kinase inhibitors, and immunotherapy, offer potential alternative strategies for managing primary hepatic SCC.

In our case, the patient died 5 months after the diagnosis without treatment.

Even with the application of treatment strategies proven effective in other liver malignancies, disease recurrence continues to be a major challenge. To develop effective therapeutic strategies and improve patient outcomes, further investigation is essential to gain a comprehensive understanding of the molecular mechanisms underpinning this malignancy.

## 8. Conclusions

Primary SCC of the liver is a rare malignancy with a poor prognosis. Early diagnosis is difficult, and treatment options remain limited. Further research is needed to clarify its pathogenesis and improve survival outcomes.

## Figures and Tables

**Table 1:** Laboratory data on admission.

<Biochemistry>		<Complete Blood Count>		<Hepatitis Virus Markers>	
TP	6.4 g/dL	WBC	77 × 10^2^/µL	HCV Ab	(−)
Alb	2.9 g/dL	RBC	427 × 10^4^/µL	HBs Ag	(−)
AST	40 U/L	Hb	12.4 g/dL		
ALT	26 U/L	Plt	19.3 × 10^4^/µL	<Tumor markers>	
ALP	70 U/L			AFP	6.1 ng/mL
ɤ-GTP	88 U/L	<Blood sugar>		PIVKA-II	36 mAU/mL
T-Bil	0.4 mg/dL	Glu	130 mg/dL	CA19-9	12.7 U/mL
LDH	298 U/L	HbAlc	6.0%	CEA	2.1 ng/mL
AMY	76 U/L			SCC	1.0 ng/mL
BUN	6.0 mg/dL			CYFRA	2.5 ng/mL
Cre	0.68 mg/dL			PSA	4.558 ng/mL

## Data Availability

The original contributions presented in this study are included in the article. Further inquiries can be directed to the corresponding author.
